# Buffalo Academy of Medicine

**Published:** 1903-05

**Authors:** F. W. McGuire


### SOCIETY PROCEEDINGS.

### Buffalo Academy of Medicine.

*Stated Meeting, held March 31, 1903.*

REPORTED BY F. W. McGUIRE, M. D., Secretary.

THE president, Dr. HERMAN E. HAYD, called the members to order at 9 p.m.

The minutes of the last stated meeting, held January 20, 1903, were read and approved.

The council recommended the election of Dr. Guiseppe Tartaro, who was duly balloted for and elected a member of the Academy.

Nomination of officers resulted as follows: president, Dr. Joseph W. Grosvenor; secretary, Dr. Edwin A. Bowerman; treasurer, Dr. Charles S. Jewett; and trustee for three years Dr. L. Schroeter.

The program of the evening was furnished by the section on ophthalmology, which consisted of a paper read by Dr. FRANK WHITEHILL HINKEL, entitled

A CONSIDERATION OF THE VALUE OF TOPICAL APPLICATIONS TO THE UPPER AIR TRACT.

It was discussed by Drs. Blaauw, Urban, Snow, Benedict and Hinkel.

The President appointed the following committee, composed of Charles S. Jewett, Thomas F. Dwyer, and J. H. Dowd, to prepare suitable resolutions regarding the death of Dr. William C. Fritz.

The chairman of the committee, GROVER W. WENDE, appointed to present resolutions regarding the death of Dr. Jacob M. Kraus, offered the following memorial:

The Buffalo Academy of Medicine hereby records its profound sorrow at the death of a fellow member, Dr. Jacob M. Kraus, which occurred on the 19th day of December, 1902.

Dr. Kraus was born in Clarence, N.Y., in March, 1866. He was a graduate of Parker's Union School, in his native town; he then studied medicine and was graduated from the University of Buffalo with the class of 1889. After two years of surbordinate service in the hospital connected with the Erie County Almhouse, Dr. Kraus was appointed to the position of house physician which he continued to hold until entering upon private practice.

He was specially interested in diseases of the skin and at the time of his death held the position of dermatologist, both to the

Buffalo German Hospital and the German Dispensary. He was a member of the New York State Medical Association, the Erie County Medical Association, the Roswell Park Medical Club, and was also a fellow of the Buffalo Academy of Medicine.

Dr. Kraus became a physician from the very love of medical science and was intelligent, conscientious, painstaking and progressive. The profession had in him one of its most honorable and useful members. He was honest and high-minded to a degree. In private life he was a modest gentleman and possessed in general combination the best qualities of a citizen. The sincerest sympathy of the Academy is hereby extended to his sorrowing family and it is ordered that this action be entered in its minutes.

GROVER WENDE, M. D.,
WILLIAM MEISBERGER, M. D.,
JOHN A. PETTIT, M. D.

It was moved and seconded that the memorial be adopted and spread on the minutes and a copy sent to the family. The chairman of the committee, Dr. Wm. C. Krauss, presented the following memorial of Dr. Herman Mynter:

WHEREAS, In the death of Dr. Herman Mynter, The Buffalo Academy of Medicine loses a former president and one of its most ardent workers; and,

WHEREAS, his zeal and energy coupled with his scientific attainments made him one of its most valued members. Therefore be it

*Resolved,* That the Buffalo Academy of Medicine tenders to his afflicted family its sincere sympathy, that this resolution be transmitted to them, and a copy be spread upon the minutes of the Academy.

WM. C. KRAUSS, M. D.,
EUGENE A. SMITH, M. D.,
L. SCHROETER, M. D.

Dr. M. D. MANN gave notice in writing to amend Article III. of the by-laws by adding the words "or hold office" after the words "to vote" in the last line.

Adjourned at 10 p.m. Attendance, 35.

